# Supplementary material for: Transcriptomic Evidence for Cell-Autonomous Sex Differentiation of the Gynandromorphic Fat Body in the Silkworm, Bombyx mori
Source: J Dev Biol. 2024 Nov 20;12(4):31. doi: 10.3390/jdb12040031 (PMC11587106; doi:10.3390/jdb12040031)
Supplement: Supplementary file 1 [file jdb-12-00031-s001.zip › JDB_MGSuzuki_TableS1.pdf]

Table S1. Primer sequences and PCR conditions used for RT-PCR

Target gene	Primers	Sequence (5'→3')	Denaturation	Annealing	Elongation	N°cycles
Bmdsx	BmdsxF2	CGCCTTACCGCAGACAGGCAG	98℃	57℃	72℃	35
	BmdsxR4	GCGCAGTGTCGTCGCTACAAGG	10 s	30 s	60 s	
