# Supplementary material for: Transcriptomic Evidence for Cell-Autonomous Sex Differentiation of the Gynandromorphic Fat Body in the Silkworm, Bombyx mori
Source: J Dev Biol. 2024 Nov 20;12(4):31. doi: 10.3390/jdb12040031 (PMC11587106; doi:10.3390/jdb12040031)
Supplement: Supplementary file 1 [file jdb-12-00031-s001.zip › JDB_MGSuzuki_TableS2.pdf]

Table S2. Primer sequences used for qRT-PCR

Target gene	Primers	Sequence (5'→3')
Fem	Fem qrt F1	ACCGCTTGGATCAGAAAGTG
	Fem qrt R1	GGCTTCGACGTTGTTTCATT
BmVg	BmVgF	CAGCGGAAGTGCTTTCAAAGC
	BmVgR	TTGAGATCAACAGGCAGTTCC
ImpM	IMPE7F1	ATGCGGGAAGAAGGTTTTATG
	IMPE7R1	AATGTGAACGGTGGTCACGTG
sp-1	sp1 F1	GACTCGTCGTGTAATGGAAAGC
	sp1 R1	ATGTGGGCAAGAGCATACCG
JhSP2	JhSP2 qr F1	ATGGAACGTCTGAGCGTCGG
	JhSP2 qr R1	GGCACTTCCACGCCATTGTG
