# Supplementary material for: Transcriptomic Evidence for Cell-Autonomous Sex Differentiation of the Gynandromorphic Fat Body in the Silkworm, Bombyx mori
Source: J Dev Biol. 2024 Nov 20;12(4):31. doi: 10.3390/jdb12040031 (PMC11587106; doi:10.3390/jdb12040031)
Supplement: Supplementary file 1 [file jdb-12-00031-s001.zip › Supporting information.pdf]

Supporting information

Figure S1. Mechanism of development of gynandromorphic individuals in the silkworm m042 strain. In insects, the division of an egg nucleus is arrested at metaphase during the first meiotic division. Meiosis is resumed when the sperm enters the egg, at which time the first meiotic division is immediately completed and the first polar body nucleus forms. Then, the second maturation division occurs and the second polar body nucleus is expelled. In *Bombyx mori*, degeneration of the polar body nucleus does not occur during the early cleavage stage, and polar body nuclei may be observed up until 9 h after egg lying. Insects do not have the multiple fertilization rejection mechanism observed in mammals, allowing multiple sperm to enter the egg. Heritable mosaic mutant strains, such as m042, have abnormalities in the second polar body degeneration process, and therefore the second polar body nucleus escapes degeneration at high frequency. This leads to the formation of two zygotic nuclei. When the egg nucleus has a Z chromosome, the second polar body nucleus has a W chromosome (and vice versa), resulting in the development of these two fertilized nuclei into a male–female mosaic embryo.

(PDF)

Figure S2. Gynandromorphic individuals subjected to RNA-seq analysis in this study. (**A**) Dorsal view of day-3 fifth instar gynandromorphic larvae (mos1 and mos2). Total RNA was purified separately from mos1 and mos2 fat bodies and subjected to the analysis. Scale bar: 10 mm. (**B**) To investigate the sex differential status of the fat

bodies, the expression pattern of *Bmdsx* was analyzed by RT-PCR as described in Figure 1. For comparison, total RNAs isolated from three control females and three control males were also subjected to the same RT-PCR analysis. RT-PCR products were separated by electrophoresis through 1.5% agarose gels containing ethidium bromide (1 mg/mL). Arrows to the right of the gel indicate the positions of *BmdsxF* and *BmdsxM*. (PDF)

S1 Table. Primer sequences and PCR conditions used for RT-PCR.

(DOCX)

S2 Table. Primer sequences used for qRT-PCR.

(DOCX)

S3 Table. List of S-DEGs. S-DEGs are sorted according to the degree of sex difference in the expression level (logFC Male vs Female).

(XLSX)

S4 Table. Read counts of all genes in male and female fat bodies identified by RNA-seq analysis.

(XLSX)

S5 Table. Differential expression levels (log FC Male vs Female) of all genes in male and female fat bodies identified by RNA-seq analysis.

(XLSX)
