# Supplementary figures and images for: Transcriptomic Evidence for Cell-Autonomous Sex Differentiation of the Gynandromorphic Fat Body in the Silkworm, Bombyx mori
Source: J Dev Biol. 2024 Nov 20;12(4):31. doi: 10.3390/jdb12040031 (PMC11587106; doi:10.3390/jdb12040031)

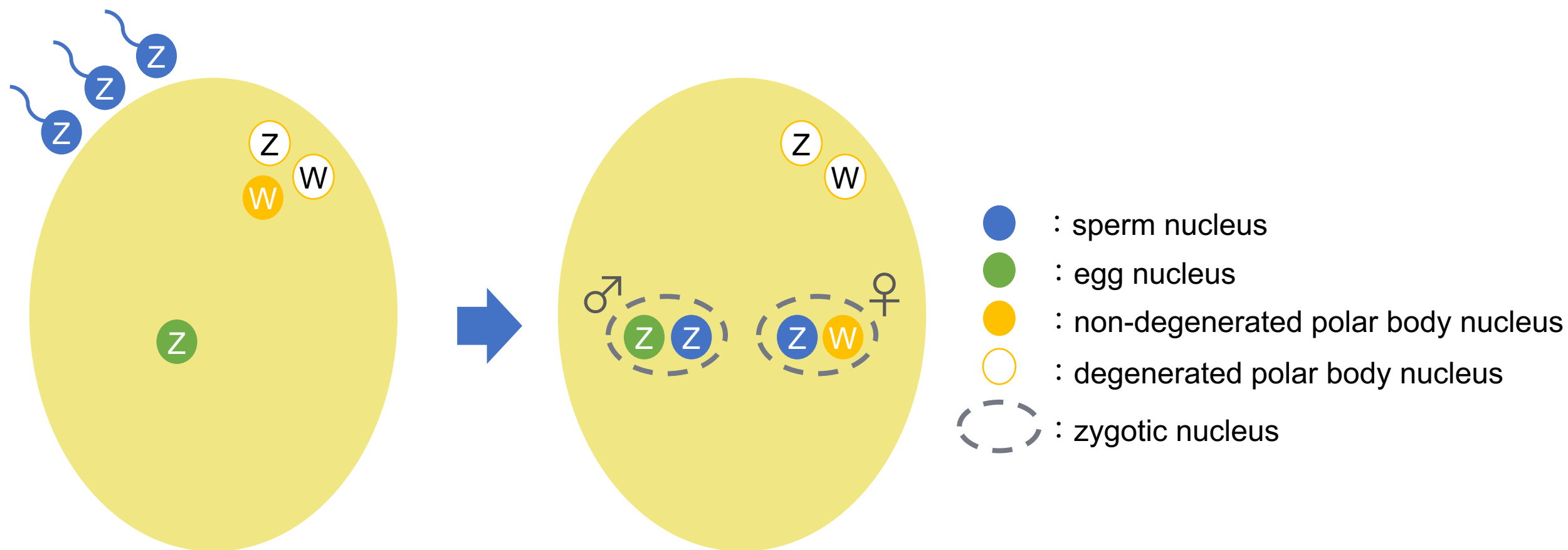

**Figure S1**

Supplement: Supplementary file 1 [file jdb-12-00031-s001.zip › MGSuzuki_FigureS1.pdf]

**A****mos1**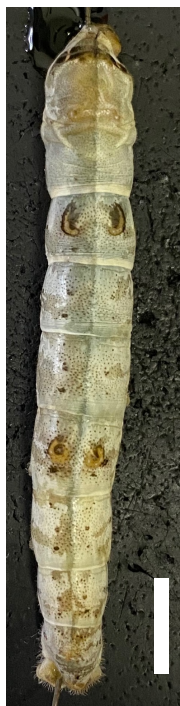**mos2**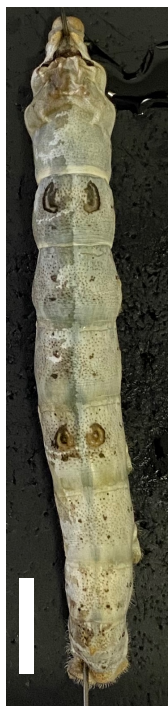**B**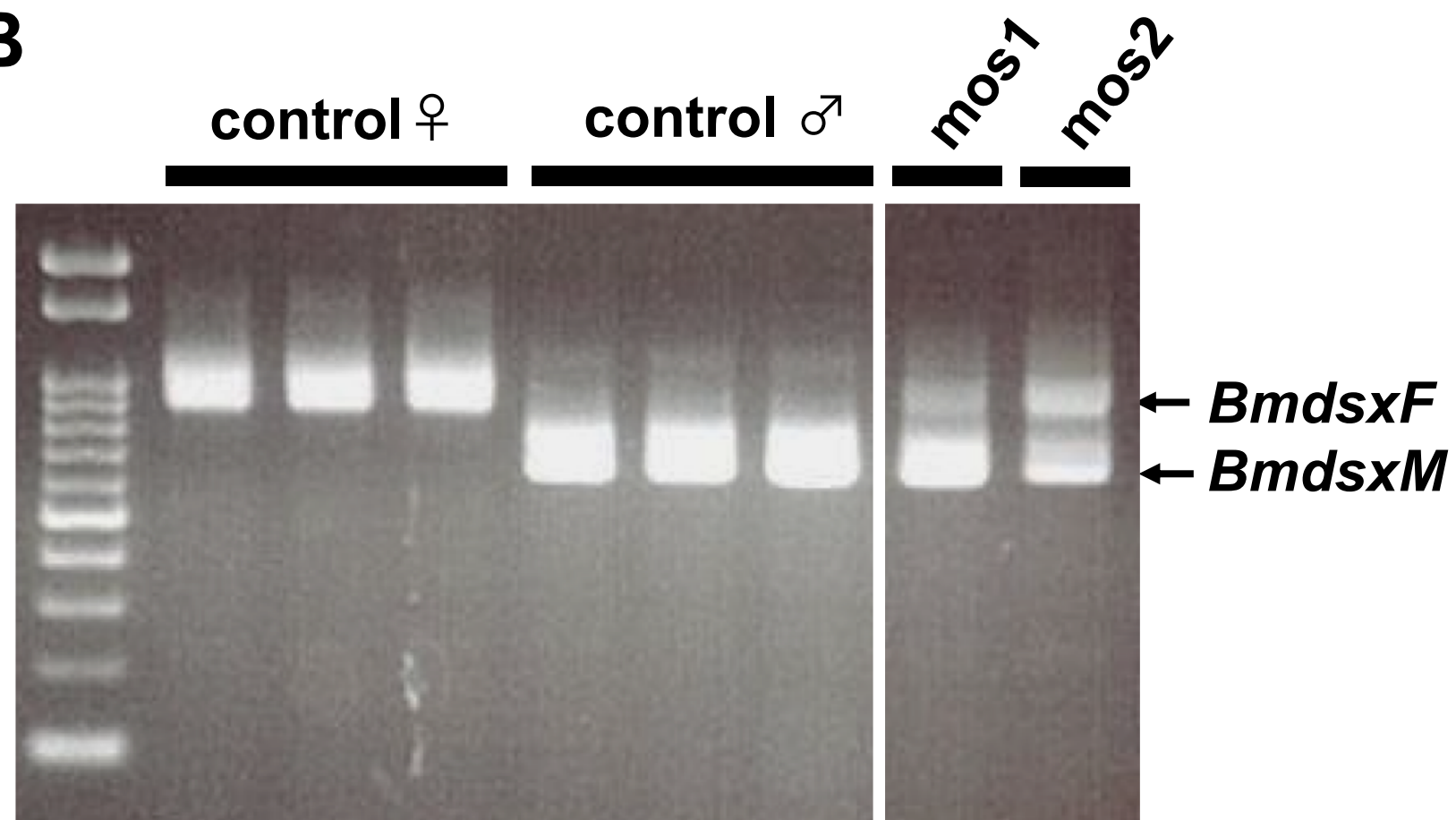

Figure S2

Supplement: Supplementary file 1 [file jdb-12-00031-s001.zip › MGSuzuki_FigureS2.pdf]
